# Supplementary material for: FAM111 protease activity undermines cellular fitness and is amplified by gain‐of‐function mutations in human disease
Source: EMBO Rep. 2020 Aug 9;21(10):e50662. doi: 10.15252/embr.202050662 (PMC7534640; doi:10.15252/embr.202050662)
Supplement: Supplementary file 1 — Appendix [file EMBR-21-e50662-s001.pdf]

**Table of contents**

**Appendix Figure S1**……....................................................................................................2

**Appendix Figure S2**…...........................................................................................................4

**Appendix Figure S3**……....................................................................................................6

**Appendix Figure S4**….........................................................................................................8

**Appendix Figure S5**……..................................................................................................10

**Appendix Figure S6**….........................................................................................................12

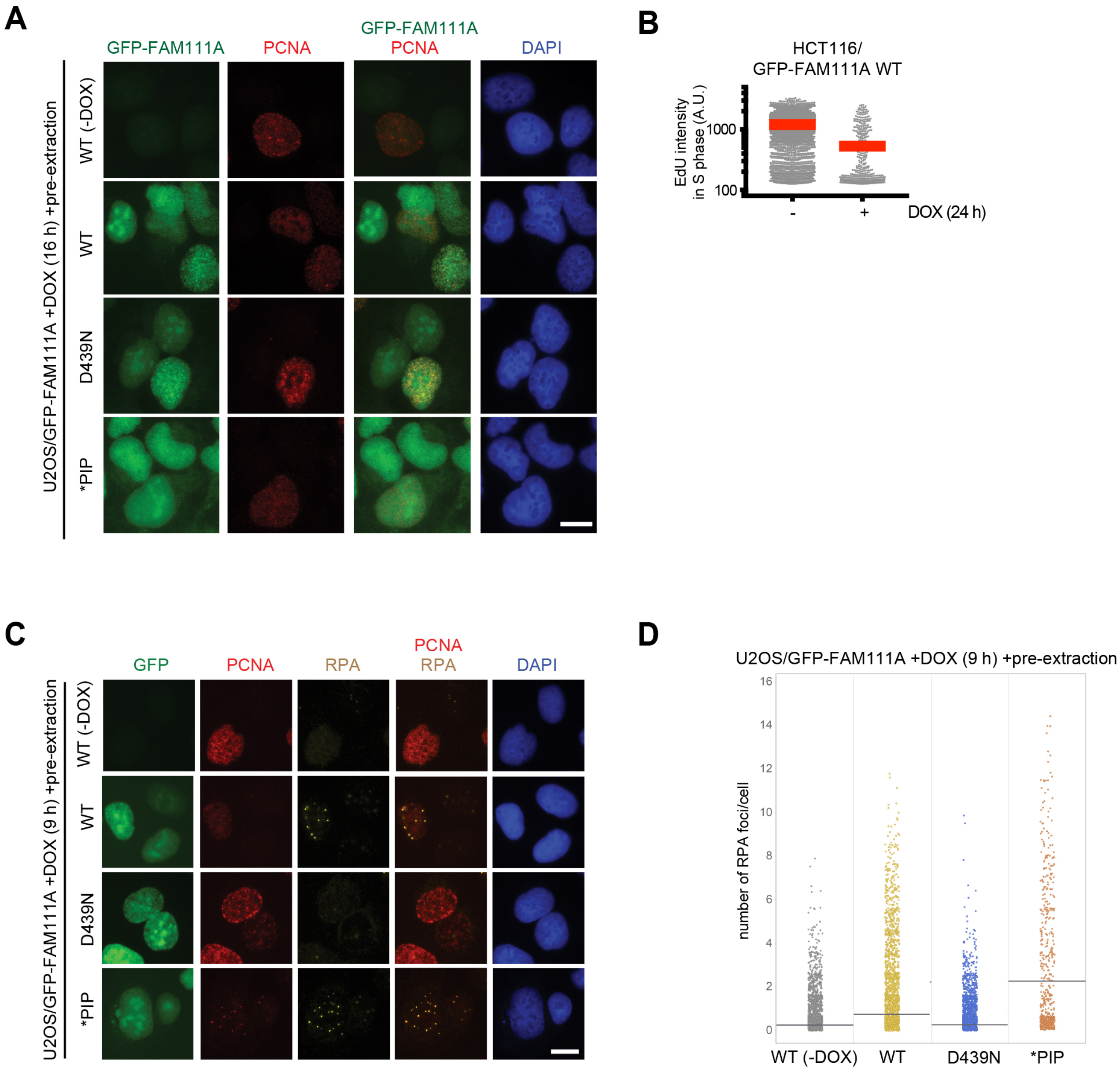

A
GFP-FAM111A
PCNA
GFP-FAM111A
PCNA
DAPI
U2OS/GFP-FAM111A +DOX (16 h) +pre-extraction
WT (-DOX)
WT
D439N
*PIP
B
HCT116/
GFP-FAM111A WT
EdU intensity
in S phase (A.U.)
1000
100
-
+
DOX (24 h)
C
GFP
PCNA
RPA
PCNA
RPA
DAPI
U2OS/GFP-FAM111A +DOX (9 h) +pre-extraction
WT (-DOX)
WT
D439N
*PIP
D
U2OS/GFP-FAM111A +DOX (9 h) +pre-extraction
number of RPA foci/cell
16
14
12
10
8
6
4
2
0
WT (-DOX)
WT
D439N
*PIP

**Appendix Figure S1.**
**Elevated FAM111A proteolytic activity impairs DNA replication and triggers replication stress**
**A.** Representative images of U2OS/GFP-FAM111A cell lines that were treated or not with DOX, fixed and stained with PCNA antibody. Mutation of the PIP box abrogates FAM111A localization to PCNA-positive DNA replication foci. **B**. DNA replication rates in HCT116/GFP-FAM111A WT cells treated or not with DOX, pulse-labeled with EdU and stained with DAPI were analyzed by quantifying EdU signal intensity in S phase cells using QIBC (*n*>500 cells per condition; red bars, mean (A.U., arbitrary units)). **C**. As in (A), except that cells were co-stained with PCNA and RPA2 antibodies. **D**. Quantification of data in (C) (grey bars, average; *n*>2000 cells per condition). Scale bars, 10 µm.
Data (**A**-**D**) are representative of three independent experiments with similar outcomes.

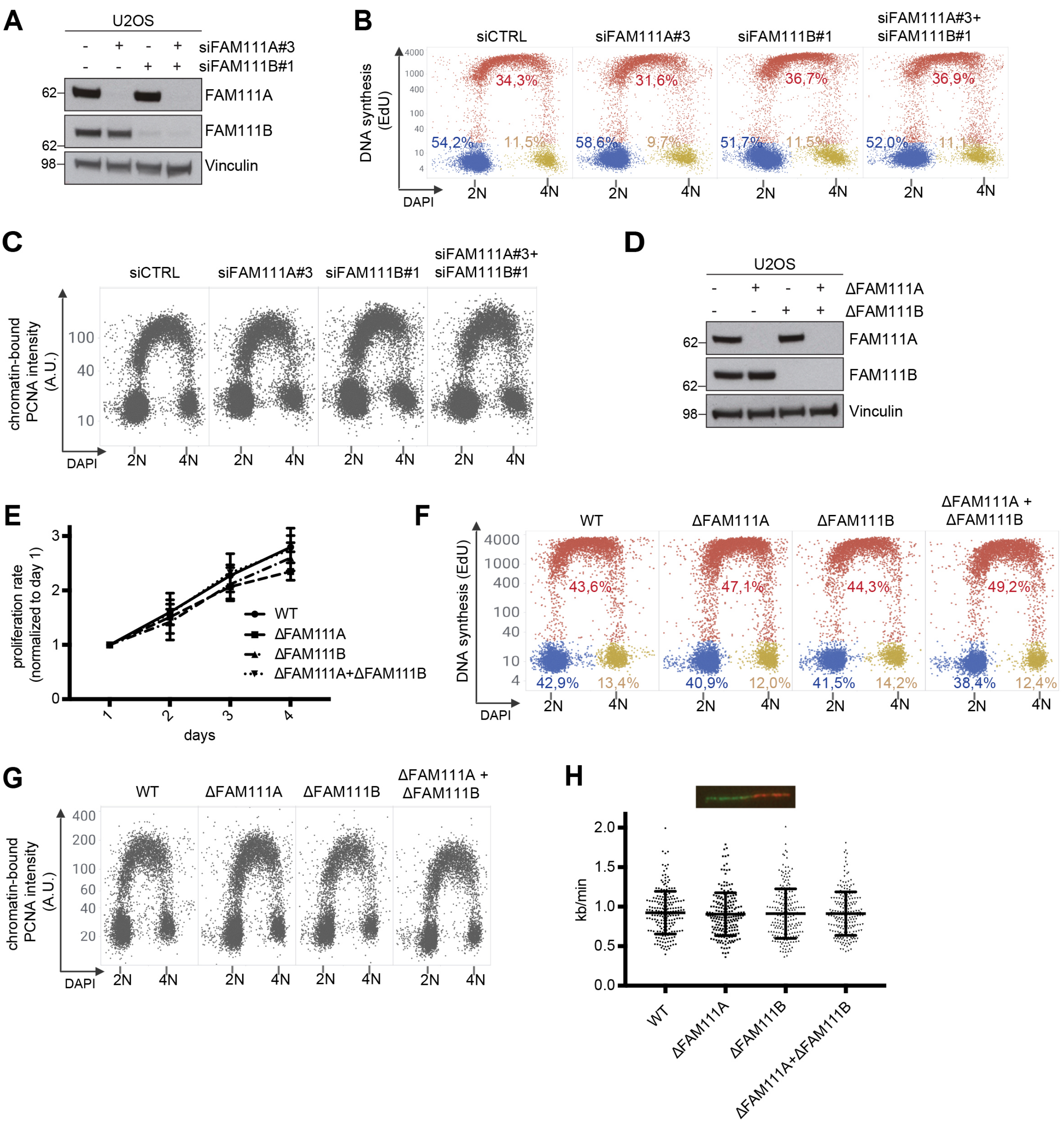
A
U2OS
siFAM111A#3
siFAM111B#1
FAM111A
FAM111B
Vinculin
B
siCTRL
siFAM111A#3
siFAM111B#1
siFAM111A#3+
siFAM111B#1
DNA synthesis (EdU)
DAPI
34,3%
31,6%
36,7%
36,9%
54,2%
11,5%
58,6%
9,7%
51,7%
11,5%
52,0%
C
chromatin-bound PCNA intensity (A.U.)
DAPI
D
U2OS
ΔFAM111A
ΔFAM111B
FAM111A
FAM111B
Vinculin
E
proliferation rate (normalized to day 1)
days
WT
ΔFAM111A
ΔFAM111B
ΔFAM111A+ΔFAM111B
F
WT
ΔFAM111A
ΔFAM111B
ΔFAM111A + ΔFAM111B
DNA synthesis (EdU)
43,6%
47,1%
44,3%
49,2%
42,9%
13,4%
40,9%
12,0%
41,5%
14,2%
38,4%
12,4%
DAPI
G
WT
ΔFAM111A
ΔFAM111B
ΔFAM111A + ΔFAM111B
chromatin-bound PCNA intensity (A.U.)
DAPI
H
kb/min
WT
ΔFAM111A
ΔFAM111B
ΔFAM111A+ΔFAM111B

**Appendix Figure S2.**
**FAM111A and FAM111B are dispensable for DNA replication and cell proliferation**
**A**. Immunoblot analysis of U2OS cells transfected with indicated siRNAs. **B**. Cells in (A) were pulse-labeled with EdU, fixed and stained with DAPI. Cells were then subjected to QIBC analysis for quantification of EdU and DAPI signal intensities ($n$>2000 cells per condition). Proportion of cells in G1 (blue), S (red) and G2/M (yellow) phases is indicated. **C**. Cells in (A) were fixed and stained with PCNA antibody and DAPI. Cells were then subjected to QIBC analysis for quantification of PCNA and DAPI signal intensities ($n$>2000 cells per condition). **D**. Immunoblot analysis of U2OS cells and derivative lines with targeted knockout of *FAM111A* and/or *FAM111B*. **E**. Proliferation of cell lines in (D) was determined by quantification of Resazurin incorporation (mean±s.d.; $n$=3 independent experiments). **F**. As in (B), but using U2OS cell lines with targeted knockout of *FAM111A* and/or *FAM111B*. **G**. As in (C), but using U2OS cell lines with targeted knockout of *FAM111A* and/or *FAM111B*. **H**. DNA fiber analysis of indicated U2OS cell lines labeled with CldU (25min; red) followed by IdU (25min; green). Fork speeds were calculated as length of labeled track divided by pulse time (black bars, median±s.d.; at least 200 fibers analysed per condition). A representative fiber image is shown.
Data (**A**-**H**) are representative of three independent experiments with similar outcomes.

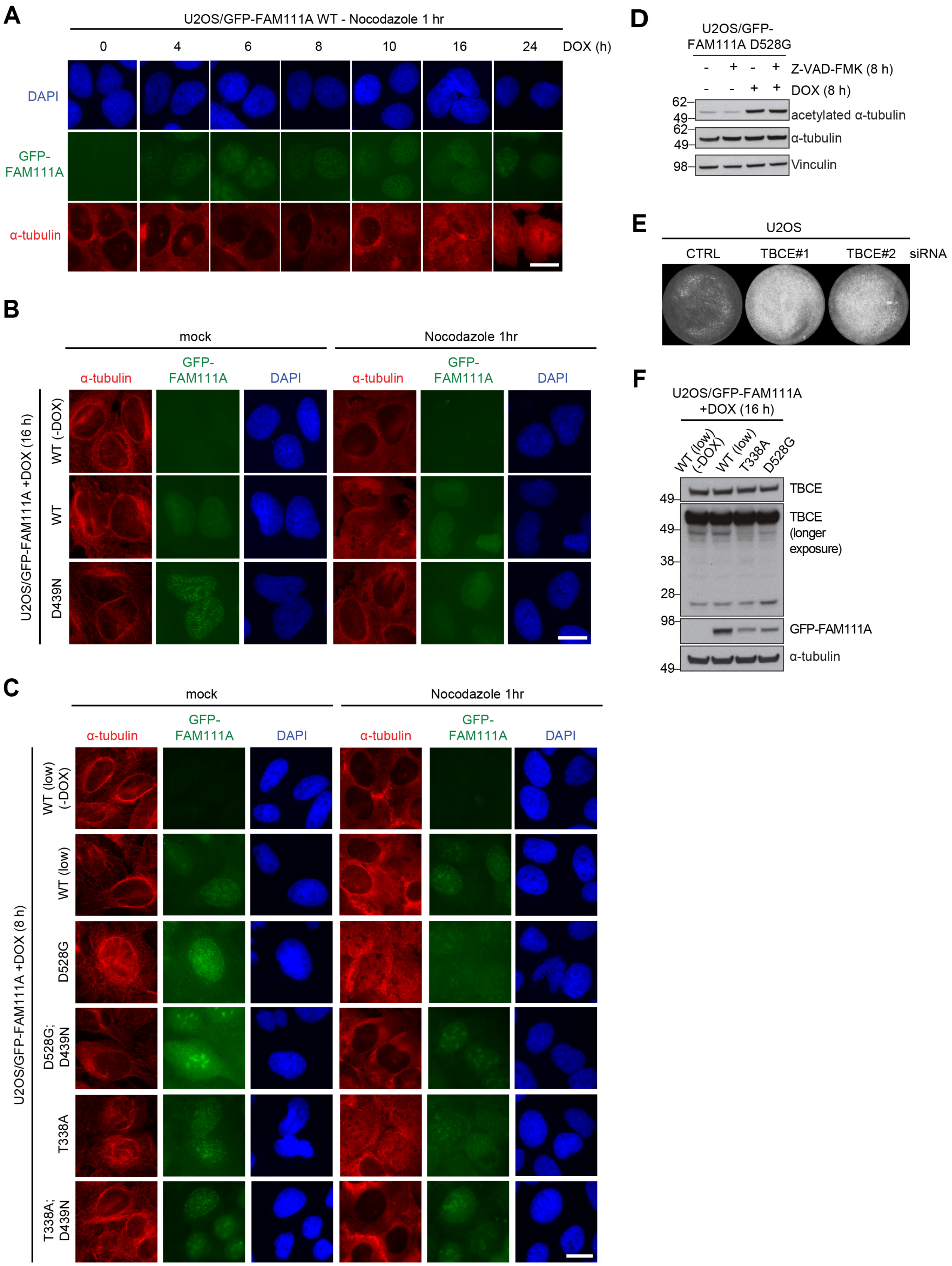
A
U2OS/GFP-FAM111A WT - Nocodazole 1 hr
0
4
6
8
10
16
24
DOX (h)
DAPI
GFP-FAM111A
α-tubulin
B
mock
Nocodazole 1hr
α-tubulin
GFP-FAM111A
DAPI
U2OS/GFP-FAM111A +DOX (16 h)
WT (-DOX)
WT
D439N
C
mock
Nocodazole 1hr
α-tubulin
GFP-FAM111A
DAPI
U2OS/GFP-FAM111A +DOX (8 h)
WT (low) (-DOX)
WT (low)
D528G
D528G; D439N
T338A
T338A; D439N
D
U2OS/GFP-FAM111A D528G
Z-VAD-FMK (8 h)
DOX (8 h)
acetylated α-tubulin
α-tubulin
Vinculin
E
U2OS
CTRL
TBCE#1
TBCE#2
siRNA
F
U2OS/GFP-FAM111A +DOX (16 h)
WT (low) (-DOX)
WT (low)
T338A
D528G
TBCE
TBCE (longer exposure)
GFP-FAM111A
α-tubulin

**Appendix Figure S3.**

**Elevated FAM111A protease activity disrupts microtubule organization**

**A**-**C**. Representative images of U2OS/GFP-FAM111A cell lines that were treated or not with DOX for the indicated times, then left untreated or exposed to nocodazole for 1 h, and fixed and stained with $\alpha$-tubulin antibody. Note that cells with elevated FAM111A protease activity display accumulation of nuclear $\alpha$-tubulin. **D**. Immunoblot analysis of U2OS/GFP-FAM111A D528G cells treated with DOX and Z-VAD-FMK for 8 h, as indicated. **E**. U2OS cells were transfected with the indicated siRNAs, fixed 4 days later and stained with crystal violet. **F**. Immunoblot analysis of U2OS/GFP-FAM111A cell lines that were left untreated or induced with DOX for 16 h. Scale bars, 10 μm.

Data are representative of three (**A**-**C**) and two (**D**-**F**) independent experiments with similar outcomes.

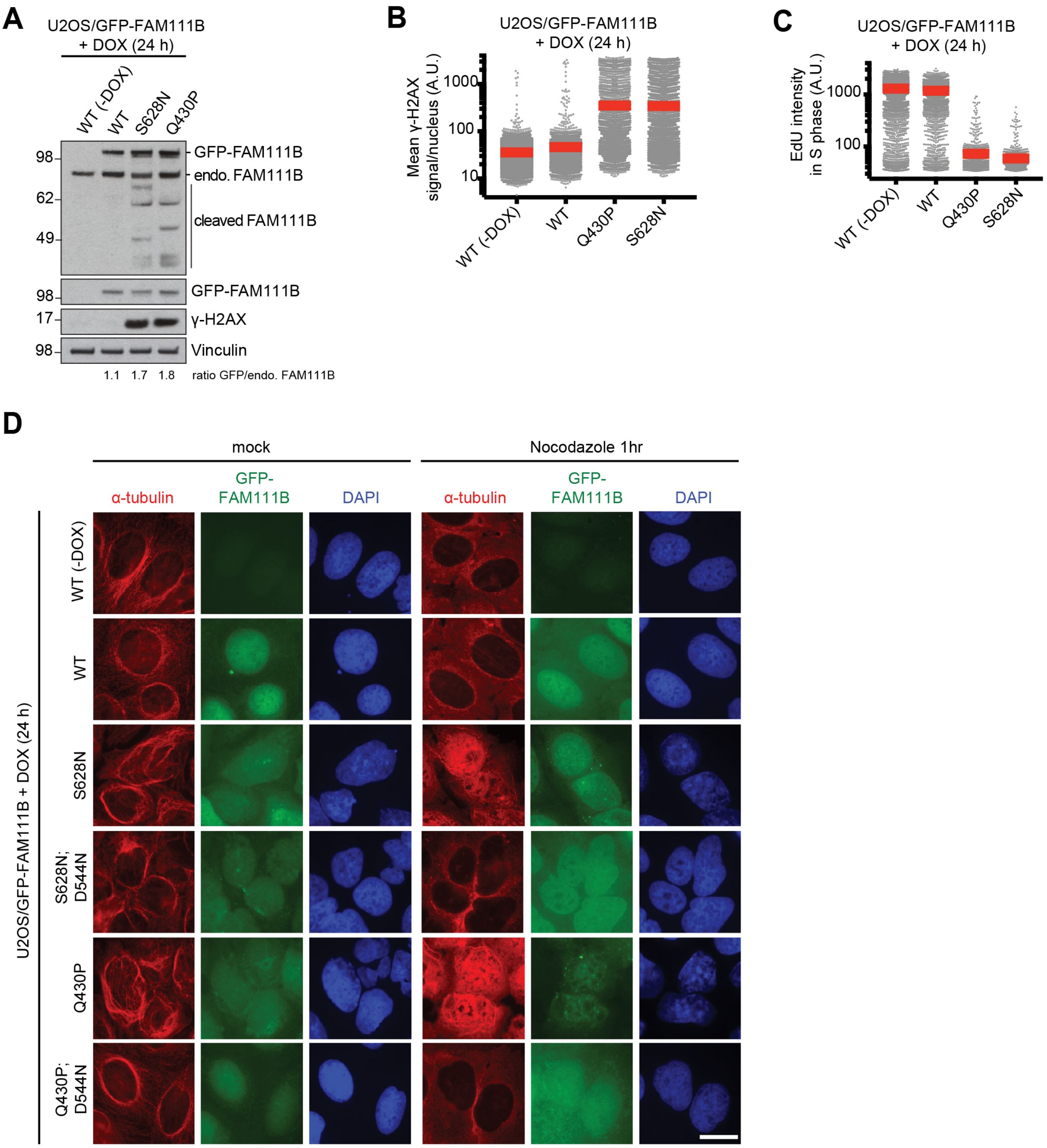
A
U2OS/GFP-FAM111B
+ DOX (24 h)
WT (-DOX)
WT
S628N
Q430P
98
62
49
GFP-FAM111B
endo. FAM111B
cleaved FAM111B
98
GFP-FAM111B
17
γ-H2AX
98
Vinculin
1.1 1.7 1.8
ratio GFP/endo. FAM111B
B
U2OS/GFP-FAM111B
+ DOX (24 h)
Mean γ-H2AX signal/nucleus (A.U.)
1000
100
10
WT (-DOX)
WT
Q430P
S628N
C
U2OS/GFP-FAM111B
+ DOX (24 h)
EdU intensity in S phase (A.U.)
1000
100
WT (-DOX)
WT
Q430P
S628N
D
mock
Nocodazole 1hr
α-tubulin
GFP-FAM111B
DAPI
U2OS/GFP-FAM111B + DOX (24 h)
WT (-DOX)
WT
S628N
S628N; D544N
Q430P
Q430P; D544N

**Appendix Figure S4.**
**Patient-associated FAM111B mutants trigger DNA replication suppression, apoptosis onset and microtubule network disruption in a protease-dependent manner**
**A**. Immunoblot analysis of U2OS/GFP-FAM111B cell lines left untreated or induced with DOX for 24 h to induce expression of WT or disease-associated GFP-FAM111B alleles. **B**. Cells treated as in (A) were fixed, stained with γ-H2AX antibody and DAPI, and γ-H2AX signal intensity was analyzed by QIBC (red bars, mean; *n*>2000 cells per condition; A.U., arbitrary units). **C**. Cells treated as in (A) were labeled with EdU, fixed and stained with DAPI. EdU signal intensity was analyzed by QIBC (red bars, mean; *n*>2000 cells per condition). **D**. Representative images of U2OS/GFP-FAM111B cell lines that were treated or not with DOX, then left untreated or exposed to nocodazole for 1 h, and fixed and stained with $\alpha$-tubulin antibody. Scale bar, 10 μm
Data (**A**-**D**) are representative of three independent experiments with similar outcomes.

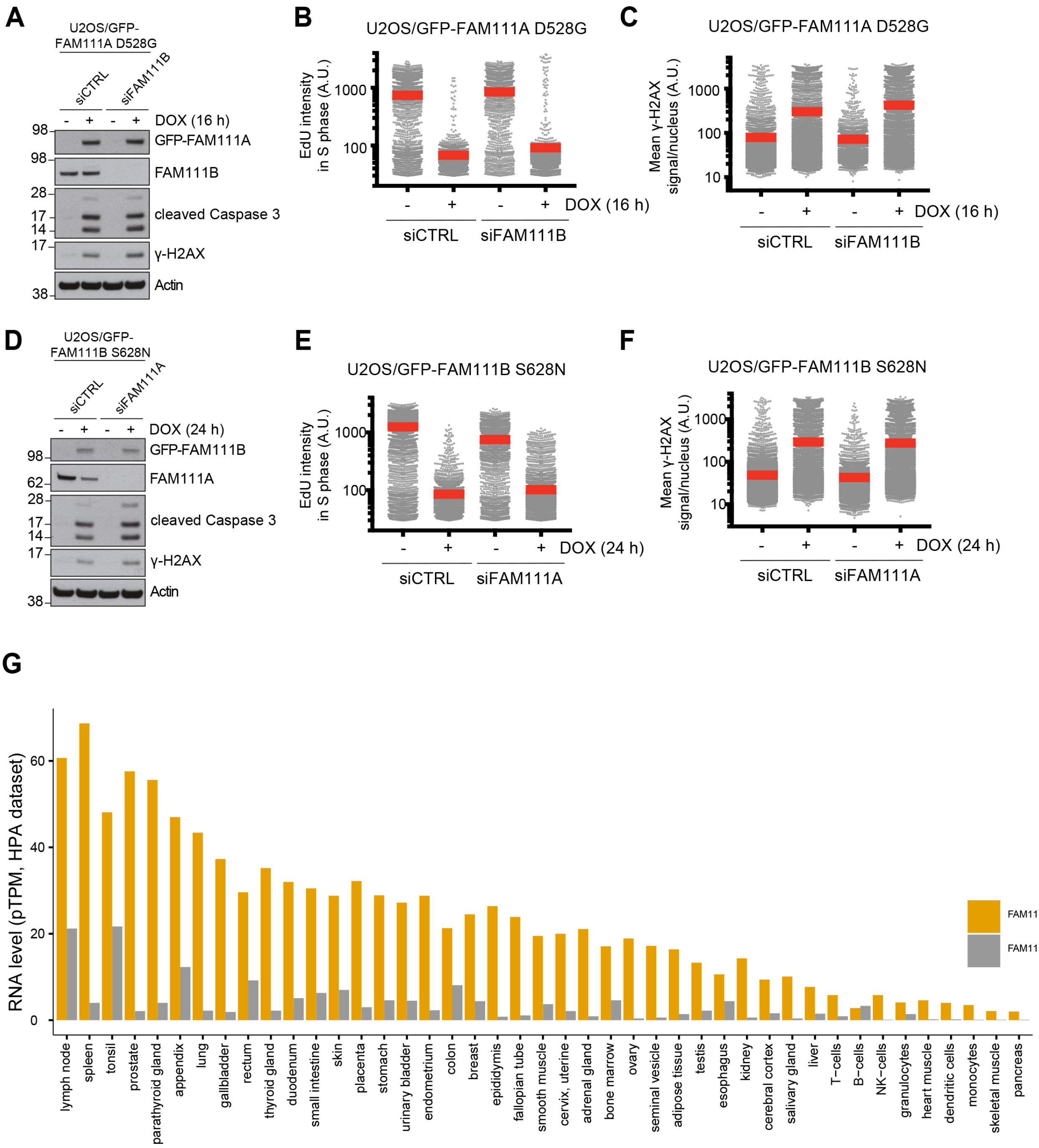
A
U2OS/GFP-FAM111A D528G
siCTRL
siFAM111B
DOX (16 h)
GFP-FAM111A
FAM111B
cleaved Caspase 3
γ-H2AX
Actin
B
U2OS/GFP-FAM111A D528G
EdU intensity in S phase (A.U.)
siCTRL
siFAM111B
C
U2OS/GFP-FAM111A D528G
Mean γ-H2AX signal/nucleus (A.U.)
D
U2OS/GFP-FAM111B S628N
siFAM111A
DOX (24 h)
GFP-FAM111B
FAM111A
E
U2OS/GFP-FAM111B S628N
F
U2OS/GFP-FAM111B S628N
G
RNA level (pTPM, HPA dataset)
FAM111A
FAM111B
lymph node
spleen
tonsil
prostate
parathyroid gland
appendix
lung
gallbladder
rectum
thyroid gland
duodenum
small intestine
skin
placenta
stomach
urinary bladder
endometrium
colon
breast
epididymis
fallopian tube
smooth muscle
cervix, uterine
adrenal gland
bone marrow
ovary
seminal vesicle
adipose tissue
testis
esophagus
kidney
cerebral cortex
salivary gland
liver
T−cells
B−cells
NK−cells
granulocytes
heart muscle
dendritic cells
monocytes
skeletal muscle
pancreas

**Appendix Figure S5.**
**FAM111A and FAM111B are differentially expressed in human tissues and impact DNA replication and apoptosis independently of each other**
**A**. Immunoblot analysis of U2OS/GFP-FAM111A D528G cells transfected with control (CTRL) or FAM111B siRNAs, and subsequently left untreated or exposed to DOX. **B**. Cells treated as in (A) were pulse-labeled with EdU, stained with DAPI and analyzed for DAPI and EdU signal intensity using QIBC (*n*>2000 cells per condition; A.U., arbitrary units). **C**. Cells treated as in (A) were fixed, stained with γ-H2AX antibody and DAPI, and γ-H2AX signal intensity was analyzed by QIBC (red bars, mean; *n*>2000 cells per condition). **D**. Immunoblot analysis of U2OS/GFP-FAM111B S628N cells transfected with control (CTRL) or FAM111A siRNAs, and subsequently left untreated or exposed to DOX. **E**. Cells treated as in (D) were pulse-labeled with EdU, stained with DAPI and analyzed for DAPI and EdU signal intensity using QIBC (*n*>2000 cells per condition). **F**. Cells treated as in (D) were fixed, stained with γ-H2AX antibody and DAPI, and γ-H2AX signal intensity was analyzed by QIBC (red bars, mean; *n*>2000 cells per condition). **G**. Human Protein Atlas RNA-seq data on *FAM111A* and *FAM111B* transcript expression levels in 37 human tissues (pTPM, protein transcripts per million).
Data (**A**-**F**) are representative of three independent experiments with similar outcomes.

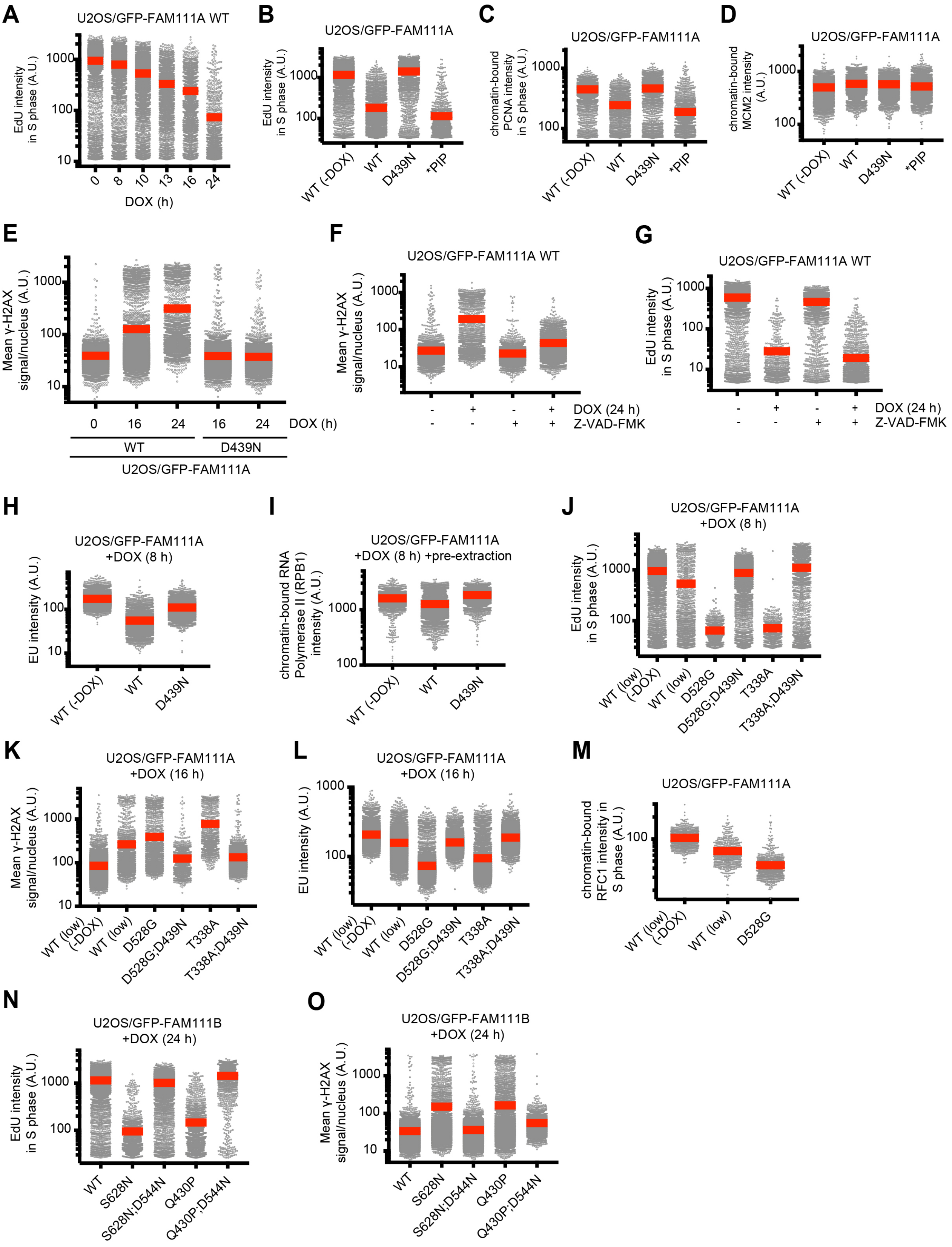
A
U2OS/GFP-FAM111A WT
EdU intensity in S phase (A.U.)
1000
100
10
0
8
10
13
16
24
DOX (h)
B
U2OS/GFP-FAM111A
EdU intensity in S phase (A.U.)
1000
100
WT (-DOX)
WT
D439N
*PIP
C
U2OS/GFP-FAM111A
chromatin-bound PCNA intensity in S phase (A.U.)
1000
100
WT (-DOX)
WT
D439N
*PIP
D
U2OS/GFP-FAM111A
chromatin-bound MCM2 intensity (A.U.)
1000
100
WT (-DOX)
WT
D439N
*PIP
E
Mean γ-H2AX signal/nucleus (A.U.)
1000
100
10
0
16
24
16
24
DOX (h)
WT
D439N
U2OS/GFP-FAM111A
F
U2OS/GFP-FAM111A WT
Mean γ-H2AX signal/nucleus (A.U.)
1000
100
10
- + - +
DOX (24 h)
- - + +
Z-VAD-FMK
G
U2OS/GFP-FAM111A WT
EdU intensity in S phase (A.U.)
1000
100
10
- + - +
DOX (24 h)
- - + +
Z-VAD-FMK
H
U2OS/GFP-FAM111A
+DOX (8 h)
EU intensity (A.U.)
100
10
WT (-DOX)
WT
D439N
I
U2OS/GFP-FAM111A
+DOX (8 h) +pre-extraction
chromatin-bound RNA Polymerase II (RPB1) intensity (A.U.)
1000
100
WT (-DOX)
WT
D439N
J
U2OS/GFP-FAM111A
+DOX (8 h)
EdU intensity in S phase (A.U.)
1000
100
WT (low) (-DOX)
WT (low)
D528G
D528G;D439N
T338A
T338A;D439N
K
U2OS/GFP-FAM111A
+DOX (16 h)
Mean γ-H2AX signal/nucleus (A.U.)
1000
100
10
WT (low) (-DOX)
WT (low)
D528G
D528G;D439N
T338A
T338A;D439N
L
U2OS/GFP-FAM111A
+DOX (16 h)
EU intensity (A.U.)
1000
100
WT (low) (-DOX)
WT (low)
D528G
D528G;D439N
T338A
T338A;D439N
M
U2OS/GFP-FAM111A
chromatin-bound RFC1 intensity in S phase (A.U.)
100
WT (low) (-DOX)
WT (low)
D528G
N
U2OS/GFP-FAM111B
+DOX (24 h)
EdU intensity in S phase (A.U.)
1000
100
WT
S628N
S628N;D544N
Q430P
Q430P;D544N
O
U2OS/GFP-FAM111B
+DOX (24 h)
Mean γ-H2AX signal/nucleus (A.U.)
1000
100
10
WT
S628N
S628N;D544N
Q430P
Q430P;D544N

**Appendix Figure S6.**

**Independent replicates of quantitative image-based cytometry (QIBC) experiments**

**A**. Biological replicate of Figure 2B (red bars, mean (A.U., arbitrary units); *n*>2000 cells per condition). **B**. Biological replicate of Figure 2D (red bars, mean; *n*>2000 cells per condition). **C**. Biological replicate of Figure 2E (red bars, mean; *n*>2000 cells per condition). **D**. Biological replicates of Figure 2F (red bars, mean; *n*>2000 cells per condition). **E**. Biological replicate of Figure 3C (red bars, mean; *n*>2000 cells per condition). **F**. Biological replicate of Figure EV3B (*n*>2000 cells per condition; red bars, mean). **G**. Biological replicate of Figure EV3E (*n*>1000 cells per condition). **H**. Biological replicate of Figure 3G (red bars, mean; *n*>2000 cells per condition). **I**. Biological replicate of Figure 3I (red bars, mean; *n*>2000 cells per condition). **J**. Biological replicate of Figure 4D (red bars, mean; *n*>2000 cells per condition). **K**. Biological replicate of Figure EV4I (red bars, mean; *n*>2000 cells per condition). **L**. Biological replicate of Figure 4F (red bars, mean; *n*>2000 cells per condition). **M**. Biological replicate of Figure EV4J (red bars, mean; *n*>2000 cells per condition). **N**. Biological replicate of Figure 5G (red bars, mean; *n*>2000 cells per condition). **O**. Biological replicate of Figure 5K (red bars, mean; *n*>2000 cells per condition).
