# Supplementary material for: FAM111 protease activity undermines cellular fitness and is amplified by gain‐of‐function mutations in human disease
Source: EMBO Rep. 2020 Aug 9;21(10):e50662. doi: 10.15252/embr.202050662 (PMC7534640; doi:10.15252/embr.202050662)
Supplement: Supplementary file 2 — Expanded View Figures PDF [file EMBR-21-e50662-s002.pdf]

Expanded View Figures

Figure EV1. FAM111 sequence conservation and recombinant proteins.

A Sequence alignment of the serine protease domain-containing portions of FAM111A proteins from different mammals. Patient-associated mutations in human FAM111A (blue circles) and catalytic triad residues in the protease domain (purple stars) are indicated. Red boxes denote residues conserved across all species shown (white); yellow boxes indicate conservative (red) or non-conservative (black) amino acid substitutions relative to the sequence of human FAM111A.
B As in (A), but showing sequence alignment for FAM111B proteins.
C Recombinant human FLAG-tagged FAM111A proteins purified from yeast were analyzed by Coomassie staining.
D Immunoblot analysis of recombinant FLAG-FAM111A proteins in (C).
E Recombinant human FLAG-tagged FAM111B proteins purified from yeast were analyzed by Coomassie staining.
F Purified recombinant FLAG-FAM111A proteins were incubated at indicated temperatures for 4 h in the absence or presence of the serine protease inhibitor AEBSF, and FAM111A auto-proteolytic activity was analyzed by immunoblotting with FLAG antibody.
G As in (F), but using purified recombinant FLAG-FAM111B WT protein. FAM111B auto-proteolytic activity was analyzed by immunoblotting with FAM111B antibody.

Data information: Data (C–G) are representative of two independent experiments with similar outcomes.

© 2020 The Author

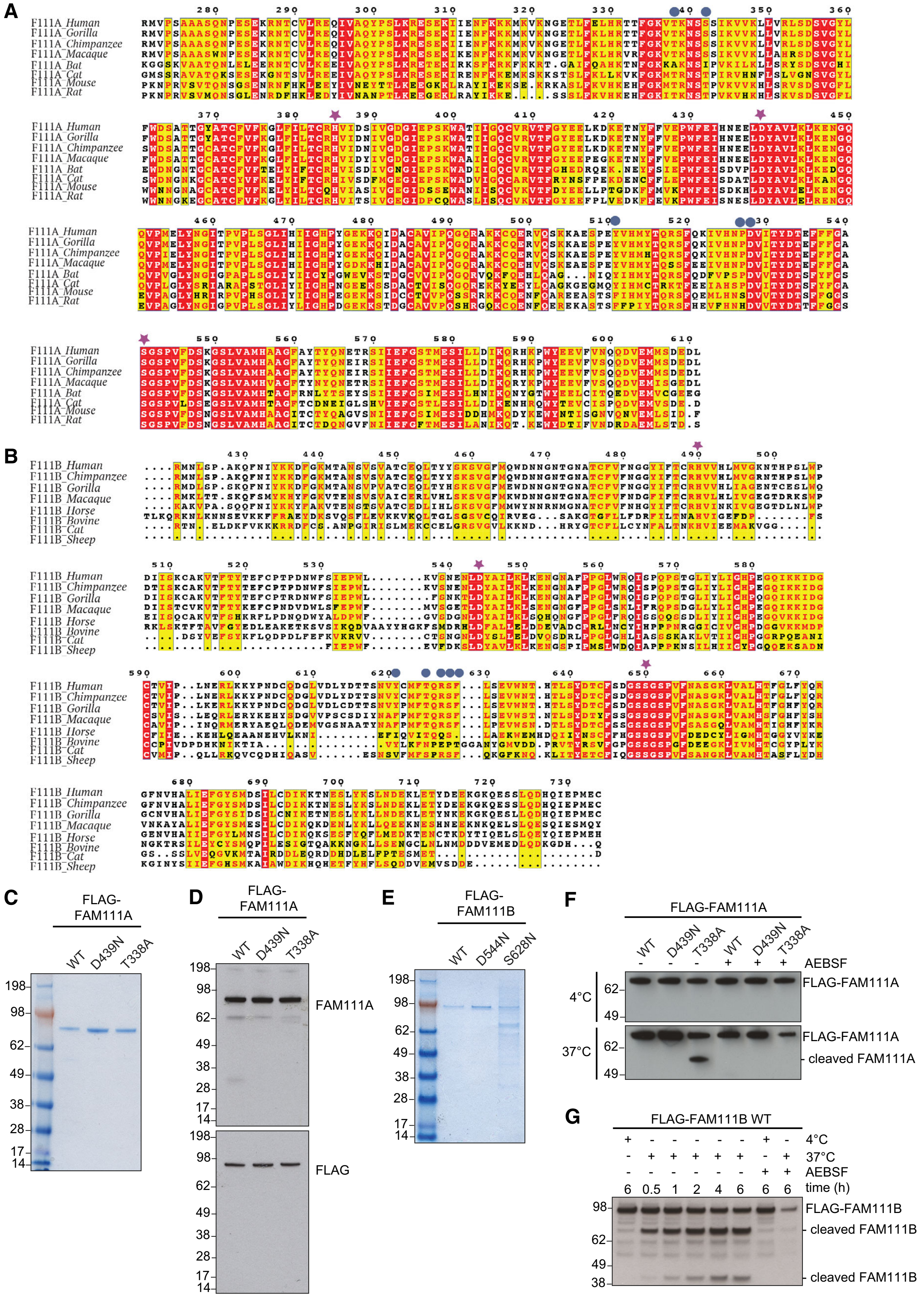

Figure EV1.

 © 2020 The Author

Figure EV2. Characterization of the interaction between FAM111A and RFC1.

A Validation of FAM111A interactors identified by mass spectrometry (Fig 2G; Dataset EV1). U2OS/GFP-FAM111A cell lines treated or not with DOX were subjected to GFP immunoprecipitation (IP) followed by immunoblotting with indicated antibodies.
B U2OS cells transfected with constructs expressing indicated FLAG-tagged RFC1 fragments were subjected to FLAG IP followed by immunoblotting with indicated antibodies.
C U2OS cells transfected with non-targeting control (CTRL) or FAM111A siRNA were pre-extracted, fixed, and stained with FAM111A antibody.
D U2OS cells labeled with EdU were pre-extracted, fixed, and stained with indicated antibodies. Endogenous FAM111A localizes to nucleoli in G1 phase and G2 phase (EdU-negative) cells and relocates to nuclear foci in S phase (EdU-positive) cells.
E U2OS cells transfected with indicated siRNAs were pre-extracted, fixed, and stained with FAM111A and RFC1 antibodies.
F Immunoblot analysis of U2OS/GFP-FAM111A cell lines treated or not with DOX. Scale bars, 10 μm.

Data information: Data (A–F) are representative of three independent experiments with similar outcomes.

© 2020 The Author

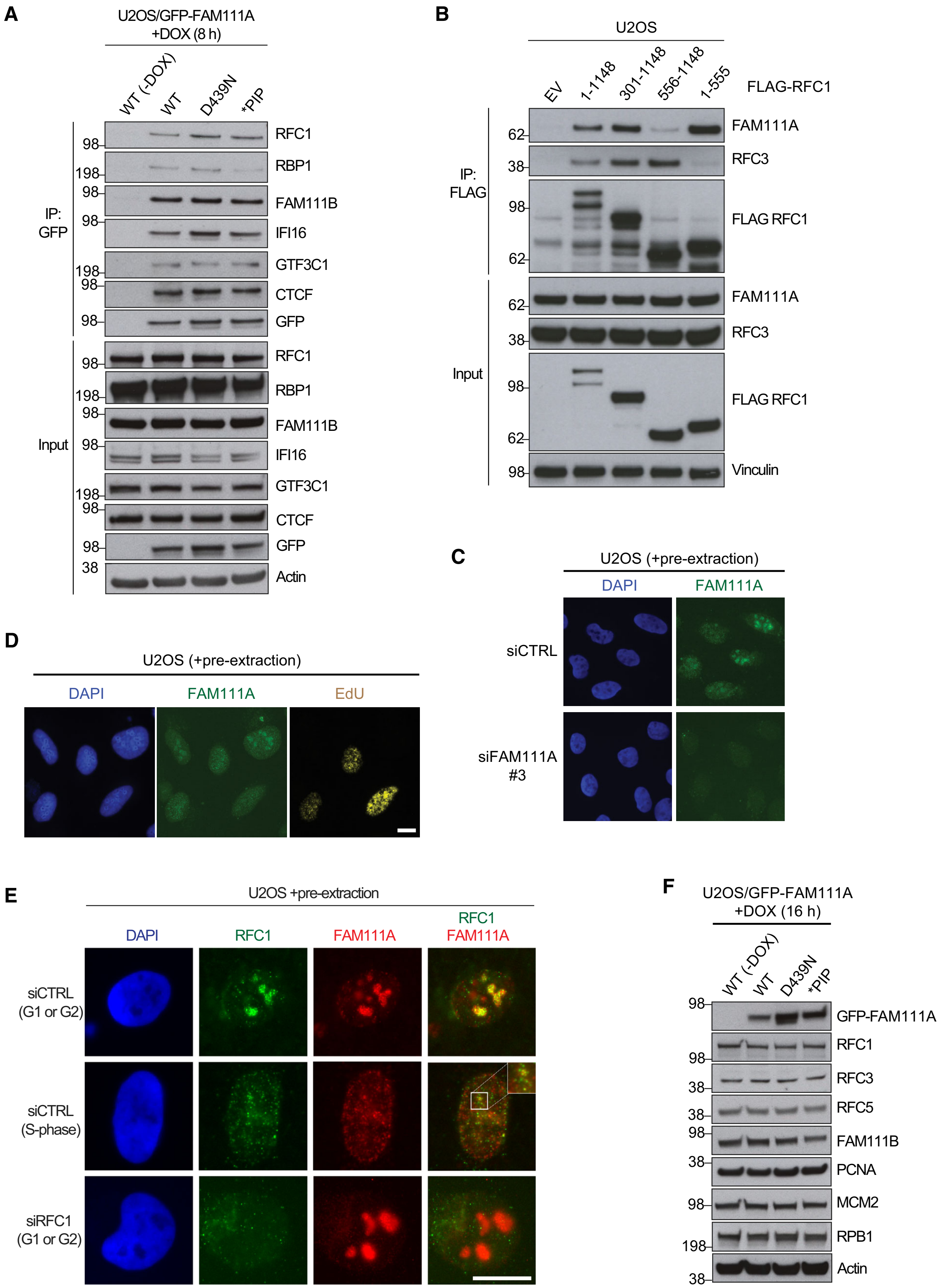

Figure EV2.

 © 2020 The Author

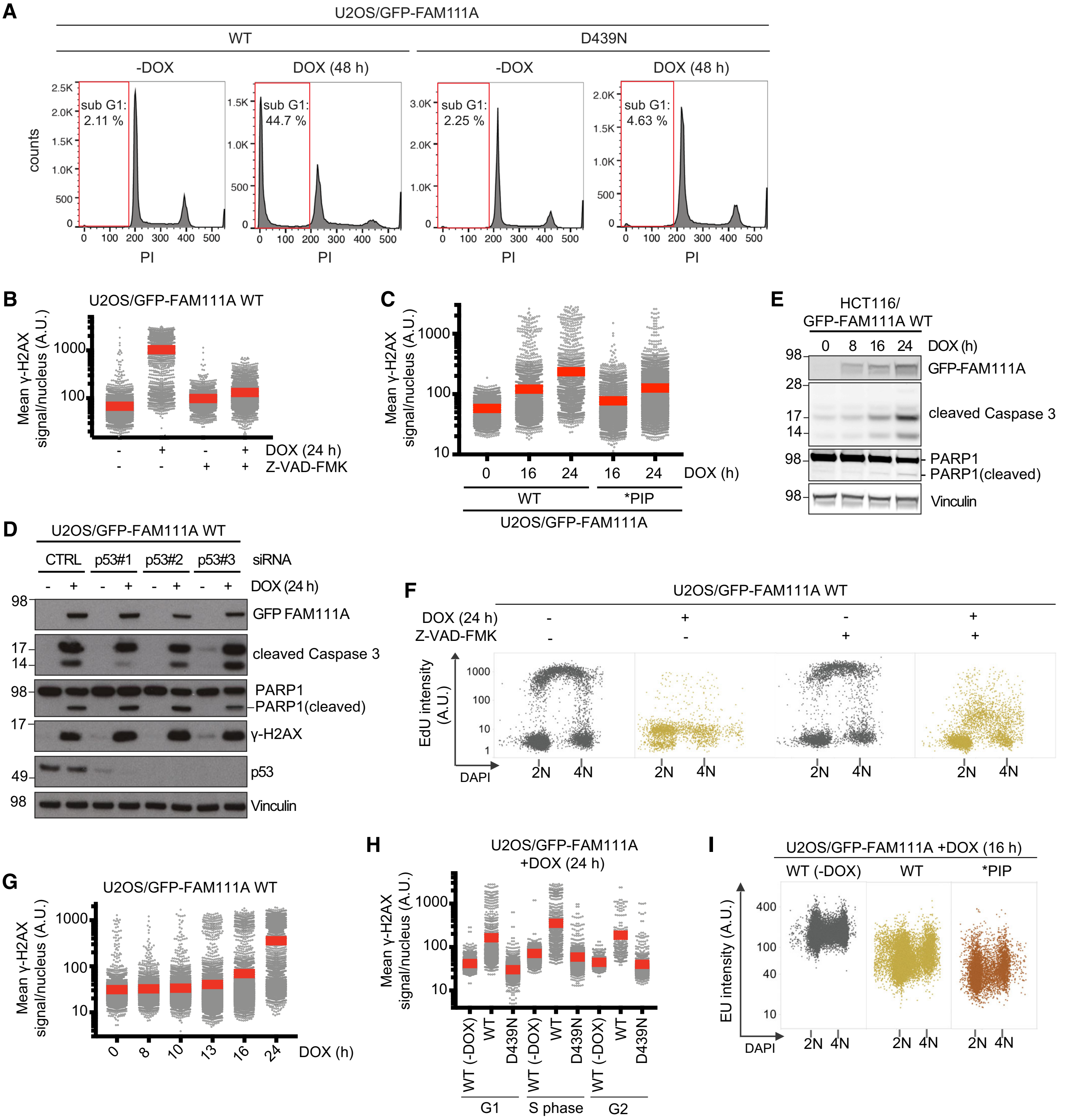
A
U2OS/GFP-FAM111A
WT
D439N
-DOX
DOX (48 h)
-DOX
DOX (48 h)
counts
sub G1: 2.11 %
sub G1: 44.7 %
sub G1: 2.25 %
sub G1: 4.63 %
PI
B
U2OS/GFP-FAM111A WT
Mean γ-H2AX signal/nucleus (A.U.)
DOX (24 h)
Z-VAD-FMK
C
0 16 24 16 24 DOX (h)
WT *PIP
U2OS/GFP-FAM111A
E
HCT116/ GFP-FAM111A WT
0 8 16 24 DOX (h)
GFP-FAM111A
cleaved Caspase 3
PARP1
PARP1(cleaved)
Vinculin
D
U2OS/GFP-FAM111A WT
CTRL p53#1 p53#2 p53#3 siRNA
DOX (24 h)
GFP FAM111A
cleaved Caspase 3
PARP1
PARP1(cleaved)
γ-H2AX
p53
Vinculin
F
U2OS/GFP-FAM111A WT
DOX (24 h)
Z-VAD-FMK
EdU intensity (A.U.)
DAPI
2N 4N
G
U2OS/GFP-FAM111A WT
Mean γ-H2AX signal/nucleus (A.U.)
0 8 10 13 16 24 DOX (h)
H
U2OS/GFP-FAM111A +DOX (24 h)
Mean γ-H2AX signal/nucleus (A.U.)
WT (-DOX) WT D439N
G1 S phase G2
I
U2OS/GFP-FAM111A +DOX (16 h)
WT (-DOX) WT *PIP
EU intensity (A.U.)
DAPI
2N 4N

Figure EV3.

Figure EV3. FAM111A proteolytic activity triggers apoptosis in a Caspase-dependent but p53-independent manner.

A U2OS/GFP-FAM111A cell lines treated or not with DOX were fixed, stained with propidium iodide (PI), and analyzed by flow cytometry. Proportion of cells with sub-G1 DNA content (red gate) is indicated. Approx. 30,000 cells were analyzed per condition.

B U2OS/GFP-FAM111A WT cells treated with DOX and/or pan-Caspase inhibitor Z-VAD-FMK for 24 h as indicated were fixed and stained with γ-H2AX antibody. Cells were then subjected to QIBC analysis of γ-H2AX signal intensity (red bars, mean (A.U., arbitrary units); $n > 2,000$ cells per condition). See also Appendix Fig S6F.

C U2OS/GFP-FAM111A cell lines treated with DOX for the indicated times were fixed and stained with γ-H2AX antibody. Cells were then subjected to QIBC analysis of γ-H2AX signal intensity (red bars, mean; $n > 2,000$ cells per condition).

D U2OS/GFP-FAM111A WT cells transfected with indicated siRNAs for 48 h were treated or not with DOX for an additional 24 h were immunoblotted with indicated antibodies.

E Immunoblot analysis of HCT116/GFP-FAM111A WT cells treated with DOX for the indicated times.

F Cells treated as in (B) were labeled with EdU, fixed, and stained with DAPI. Cells were then subjected to QIBC analysis of EdU signal intensity ($n > 1,000$ cells per condition). See also Appendix Fig S6G.

G U2OS/GFP-FAM111A WT cells treated with DOX for the indicated times were fixed and stained with γ-H2AX antibody. Cells were then subjected to QIBC analysis of γ-H2AX signal intensity (red bars, mean; $n > 2,000$ cells per condition).

H U2OS/GFP-FAM111A cell lines treated or not with DOX and labeled with EdU were fixed and stained with γ-H2AX antibody. Cells were then subjected to QIBC analysis of γ-H2AX signal intensity (red bars, mean; $n > 2,000$ cells per condition). Cells were classified as being in G1, S, and G2/M phases based on DNA content and EdU signal intensity.

I U2OS/GFP-FAM111A cell lines treated or not with DOX were pulse-labeled with EU, stained with DAPI, and analyzed by QIBC ($n > 2,000$ cells per condition). Data are derived from the experiment shown in Fig 3F.

Data information: Data (A–I) are representative of three independent experiments with similar outcomes.

Figure EV4. Patient-associated mutations hyperactivate FAM111A protease activity to exacerbate its adverse impact on cellular fitness.

A Immunoblot analysis of parental U2OS cells (−) or derivative stable cell lines conditionally expressing GFP-FAM111A WT at different levels.

B Cells in (A) were treated with DOX for 16 h, pulse-labeled with EdU, fixed, and stained with DAPI. Cells were then subjected to QIBC analysis for quantification of EdU and DAPI signal intensities ($n > 2,000$ cells per condition; A.U., arbitrary units).

C As in (B), except that cells were treated with DOX for 24 h.

D Immunoblot analysis of parental U2OS cells (−) or derivative stable cell lines expressing WT or patient-associated GFP-FAM111A alleles.

E Cells in (D) were pulse-labeled with EdU, fixed, and stained with DAPI. Cells were then subjected to QIBC analysis for quantification of EdU and DAPI signal intensities ($n > 2,000$ cells per condition).

F Quantification of data in (E) (red bars, mean).

G Representative images of U2OS/GFP-FAM111A cell lines that were treated or not with DOX for the indicated times, fixed, and co-stained with PCNA and RPA2 antibodies. Scale bar, 10 μm.

H Quantification of data in (G) (gray bars, average; $n > 2,000$ cells per condition).

I U2OS/GFP-FAM111A cell lines treated or not with DOX were stained with γ-H2AX antibody and analyzed for γ-H2AX signal intensity by QIBC (red bars, mean; $n > 2,000$ cells per condition). See also Appendix Fig S6K.

J As in (I), except that cells were stained with RFC1 antibody, pre-extracted and fixed, and stained with DAPI. RFC1 signal intensity in S phase cells (gated based on DAPI signal intensity) was analyzed by QIBC (red bars, mean; $n > 2,000$ cells per condition). See also Appendix Fig S6M.

K As in (I), except that cells were stained with RPB1 antibody and analyzed for RPB1 signal intensity by QIBC (red bars, mean; $n > 2,000$ cells per condition).

L U2OS cell lines conditionally expressing untagged ectopic FAM111A alleles were treated or not with DOX for 24 h, labeled with EdU, fixed, and stained with DAPI. Cells were then subjected to QIBC analysis for quantification of EdU and DAPI signal intensities ($n > 2,000$ cells per condition).

M Immunoblot analysis of stable U2OS/FAM111A cell lines transfected or not with FAM111A siRNA targeting the 3′UTR, and subsequently treated or not with DOX to express ectopic untagged FAM111A alleles.

Data information: Data (A–M) are representative of three independent experiments with similar outcomes.

 © 2020 The Author

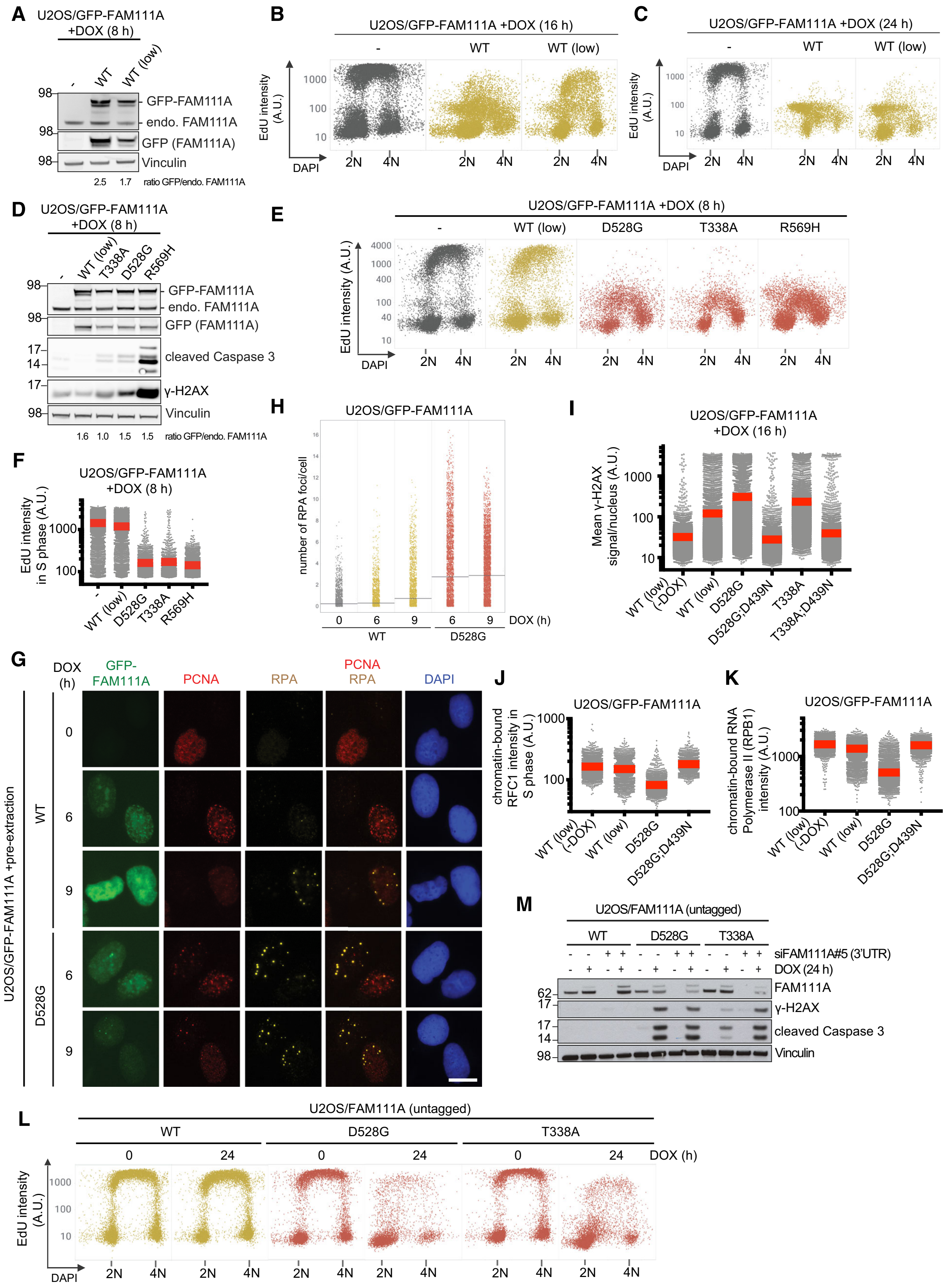
A U2OS/GFP-FAM111A +DOX (8 h)
- WT WT (low)
GFP-FAM111A
endo. FAM111A
GFP (FAM111A)
Vinculin
2.5 1.7 ratio GFP/endo. FAM111A
B U2OS/GFP-FAM111A +DOX (16 h)
C U2OS/GFP-FAM111A +DOX (24 h)
EdU intensity (A.U.)
DAPI
D U2OS/GFP-FAM111A +DOX (8 h)
- WT (low) T338A D528G R569H
cleaved Caspase 3
γ-H2AX
1.6 1.0 1.5 1.5 ratio GFP/endo. FAM111A
E U2OS/GFP-FAM111A +DOX (8 h)
F U2OS/GFP-FAM111A +DOX (8 h)
EdU intensity in S phase (A.U.)
H U2OS/GFP-FAM111A
number of RPA foci/cell
DOX (h)
I U2OS/GFP-FAM111A +DOX (16 h)
Mean γ-H2AX signal/nucleus (A.U.)
WT (low) (-DOX) WT (low) D528G D528G;D439N T338A T338A;D439N
G DOX (h) GFP-FAM111A PCNA RPA PCNA RPA DAPI
U2OS/GFP-FAM111A +pre-extraction
J U2OS/GFP-FAM111A
chromatin-bound RFC1 intensity in S phase (A.U.)
K U2OS/GFP-FAM111A
chromatin-bound RNA Polymerase II (RPB1) intensity (A.U.)
M U2OS/FAM111A (untagged)
WT D528G T338A
siFAM111A#5 (3'UTR)
DOX (24 h)
FAM111A
L U2OS/FAM111A (untagged)
WT D528G T338A
DOX (h)

Figure EV4.

Figure EV5. Patient-associated FAM111B mutants interact with and displace RFC1 and RPB1 from chromatin.

A Representative images of U2OS/GFP-FAM111B WT cells that were treated with DOX for 24 h, pulse-labeled with EdU, and stained with DAPI. No enrichment of GFP-FAM111B at EdU-positive DNA replication sites is apparent. Scale bar, 10 μm.

B U2OS/GFP-FAM111B cell lines exposed to DOX for 16 h were pulse-labeled with EdU, pre-extracted, and fixed. Cells were then stained with indicated antibodies and DAPI, and analyzed by QIBC ($n > 2{,}000$ cells per condition; A.U., arbitrary units).

C U2OS cells transfected with indicated RFC subunit expression plasmids were subjected to FLAG IP and immunoblotted with indicated antibodies.

D Parental U2OS cells or U2OS/GFP-FAM111A or FAM111B WT cell lines treated with DOX were subjected to GFP IP followed by immunoblotting with indicated antibodies.

E Representative images of U2OS/GFP-FAM111B cell lines that were treated or not with DOX, fixed, and stained with PCNA and RPA2 antibodies. Scale bar, 10 μm.

F Quantification of data in (E) (gray bars, average; $n > 2{,}000$ cells per condition).

G U2OS/GFP-FAM111B cell lines treated or not with DOX were pre-extracted, fixed, and stained with RPB1 antibody and DAPI. RPB1 signal intensity was analyzed by QIBC ($n > 2{,}000$ cells per condition).

H Quantification of data in (G) (red bars, mean; $n > 2{,}000$ cells per condition).

Data information: Data (A–H) are representative of three independent experiments with similar outcomes.

© 2020 The Author

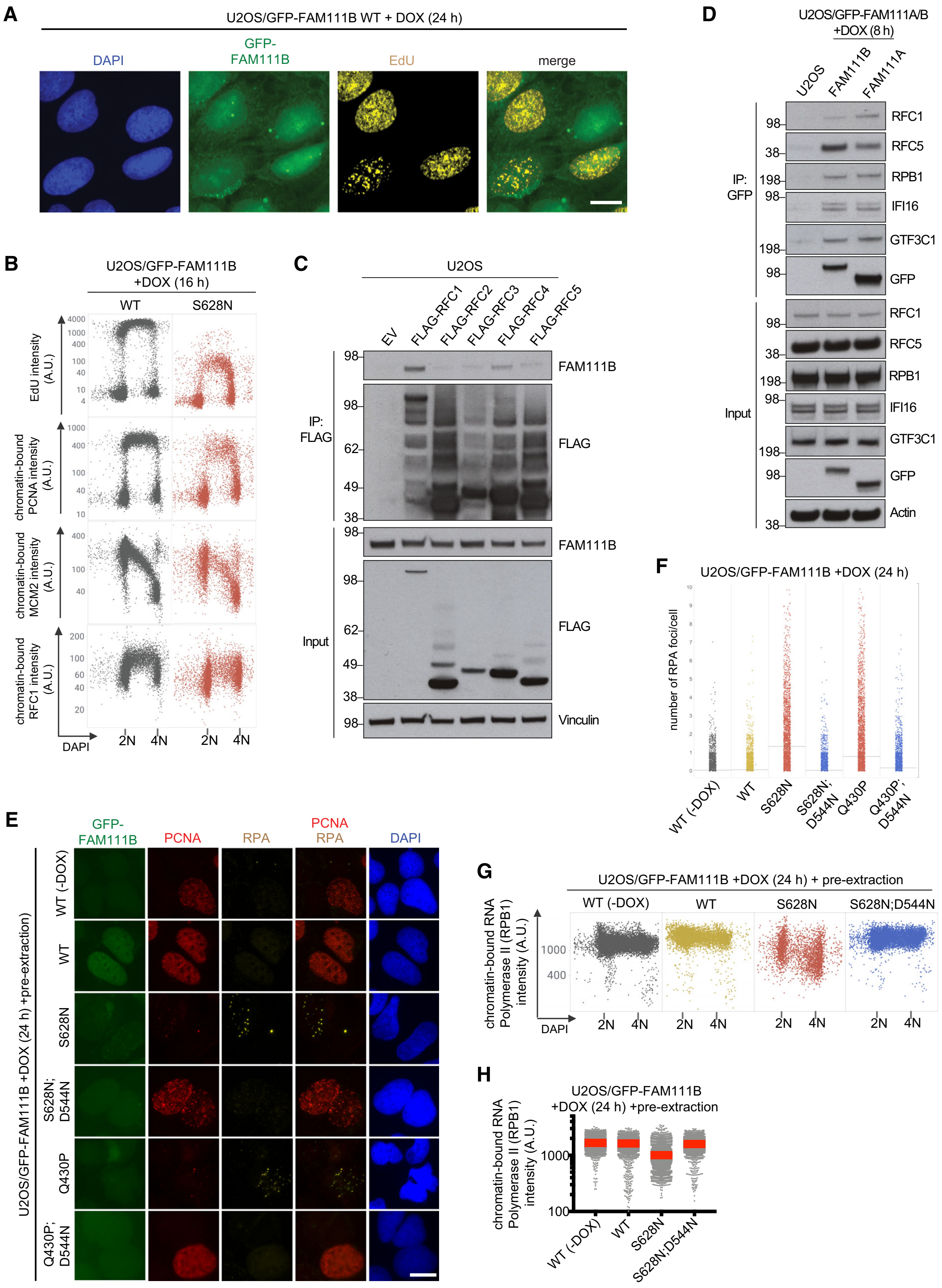
A
U2OS/GFP-FAM111B WT + DOX (24 h)
DAPI
GFP-FAM111B
EdU
merge
B
U2OS/GFP-FAM111B +DOX (16 h)
WT
S628N
EdU intensity (A.U.)
chromatin-bound PCNA intensity (A.U.)
chromatin-bound MCM2 intensity (A.U.)
chromatin-bound RFC1 intensity (A.U.)
DAPI
2N 4N
C
U2OS
EV
FLAG-RFC1
FLAG-RFC2
FLAG-RFC3
FLAG-RFC4
FLAG-RFC5
IP: FLAG
Input
FAM111B
FLAG
Vinculin
D
U2OS/GFP-FAM111A/B +DOX (8 h)
U2OS
FAM111B
FAM111A
IP: GFP
Input
RFC1
RFC5
RPB1
IFI16
GTF3C1
GFP
Actin
E
GFP-FAM111B
PCNA
RPA
PCNA RPA
DAPI
U2OS/GFP-FAM111B +DOX (24 h) +pre-extraction
WT (-DOX)
WT
S628N
S628N; D544N
Q430P
Q430P; D544N
F
U2OS/GFP-FAM111B +DOX (24 h)
number of RPA foci/cell
WT (-DOX)
WT
S628N
S628N; D544N
Q430P
Q430P; D544N
G
U2OS/GFP-FAM111B +DOX (24 h) + pre-extraction
WT (-DOX)
WT
S628N
S628N;D544N
chromatin-bound RNA Polymerase II (RPB1) intensity (A.U.)
DAPI
2N 4N
H
U2OS/GFP-FAM111B +DOX (24 h) +pre-extraction
chromatin-bound RNA Polymerase II (RPB1) intensity (A.U.)
WT (-DOX)
WT
S628N
S628N;D544N

Figure EV5.
